# Machine learning-based donor permission extraction from informed consent documents

**DOI:** 10.1186/s12859-023-05568-7

**Published:** 2023-12-15

**Authors:** Meng Zhang, Madhuri Sankaranarayanapillai, Jingcheng Du, Yang Xiang, Frank J. Manion, Marcelline R. Harris, Cooper Stansbury, Huy Anh Pham, Cui Tao

**Affiliations:** 1 McWilliam School of Biomedical Informatics, The University of Texas Health Science Center at Houston, Houston, TX 77030 USA; 2 School of Nursing, University of Michigan, Ann Arbor, MI 48104 USA; 3 Department of Artificial Intelligence and Informatics , Mayo Clinic, Jacksonville, FL 32224 USA

**Keywords:** Informed consent, Machine learning, Natural language processing, Text classification

## Abstract

**Background:**

With more clinical trials are offering optional participation in the collection of bio-specimens for biobanking comes the increasing complexity of requirements of informed consent forms. The aim of this study is to develop an automatic natural language processing (NLP) tool to annotate informed consent documents to promote biorepository data regulation, sharing, and decision support. We collected informed consent documents from several publicly available sources, then manually annotated them, covering sentences containing permission information about the sharing of either bio-specimens or donor data, or conducting genetic research or future research using bio-specimens or donor data.

**Results:**

We evaluated a variety of machine learning algorithms including random forest (RF) and support vector machine (SVM) for the automatic identification of these sentences. 120 informed consent documents containing 29,204 sentences were annotated, of which 1250 sentences (4.28%) provide answers to a permission question. A support vector machine (SVM) model achieved a F-1 score of 0.95 on classifying the sentences when using a gold standard, which is a prefiltered corpus containing all relevant sentences.

**Conclusions:**

This study provides the feasibility of using machine learning tools to classify permission-related sentences in informed consent documents.

## Background

Informed consent is a crucial component in clinical trials which encompasses a complex set of regulatory, legal, privacy and security requirements [1]. A growing number of clinical trials offer optional participation in the collection of bio-specimens for biobanking, which is so drastically different from traditional clinical trials that it has prompted an extensive debate over what needs to be ethically included in the informed consent documents [2, 3]. Biobanks are defined as “organized collections of human biological specimens comprised of cells, tissues, blood or DNA, which could be linked to clinical data and detailed individual lifestyle” [4]. The creation of biobank has greatly empowered genomic research, which relies on large samples representing a large population. This has raised concerns on the possibility of re-identification of the donor from genetic information [5]. To accommodate this research trend, on September 8, 2015, the US Department of Health and Human Services issued a notice of proposed rule making to revise certain requirements of the informed consent process under the Common Rule (45 CFR 46) [6]. One of the important provisions that were proposed is the requirement of broad consent forms for secondary, unspecified future research, even if the bio-specimen will be de-identified. Based on this provision, accurate knowledge of the intention of the person consenting to the donation and sharing of bio-specimens and associated data would have to be obtained to decide appropriately whether to share them with future researchers and entities that apply to use those resources. The overarching goal of this study was to answer the question: “given data or specimens collected as part of a biorepository, can these artifacts be distributed in line with the wishes of the specimen donors as expressed in the informed consent material and in line with constraints imposed by US law.” The ability to compare consent constraints in such a fashion while defining research cohorts is essential in large biorepositories where manually scanning thousands of consent forms is infeasible and impractical.

**Fig. 1:**
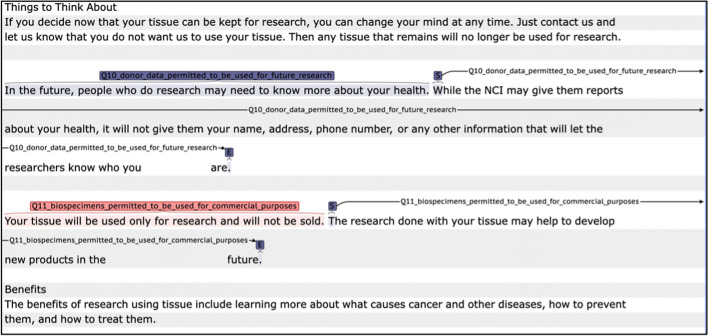
Screenshot of annotation in CLAMP

There are several challenges, however, to checking and tracking donors’ permissions on the use of their bio-specimen and data. First, no standardized template or language is available for these informed consent forms that machines can easily parse. Second, the wording in those consent forms may not be well understood by donors of what exactly they are giving permissions to [7]. Natural language processing (NLP) has shown promise in the biomedical domain in extracting information from unstructured text, but little research has been done on the application of NLP to analyze informed consent forms. Recent studies using machine learning tools are focused on developing digitalized informed consent formats, such as video, remote consent, and analyzing subject demographic and other medical elements [8]. One study developed an automatic audit system to analyze the quality of consent form itself, using Support Vector Machine [9]. No other study was found with the aim of this study, which is to develop an automatic tool to annotate informed consent documents to aid biorepository data regulation, sharing, and decision support (Fig. 1).

In this study, we evaluated machine learning algorithms for text classification using 120 informed consent documents, with particular focus on questions related to permissions, including permissions on the sharing, genetic studies, and future uses of bio-specimens and donor data. The linear support vector machine model achieved a F-1 score up to 0.95 on classifying the sentences, as the first effort of its kind, we proved the feasibility of automated annotation of informed consent documents.

## Results

### Balancing data set

**Fig. 2:**
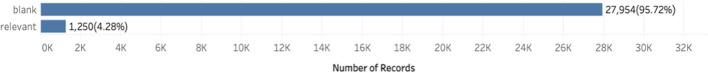
Data distribution

**Fig. 3:**
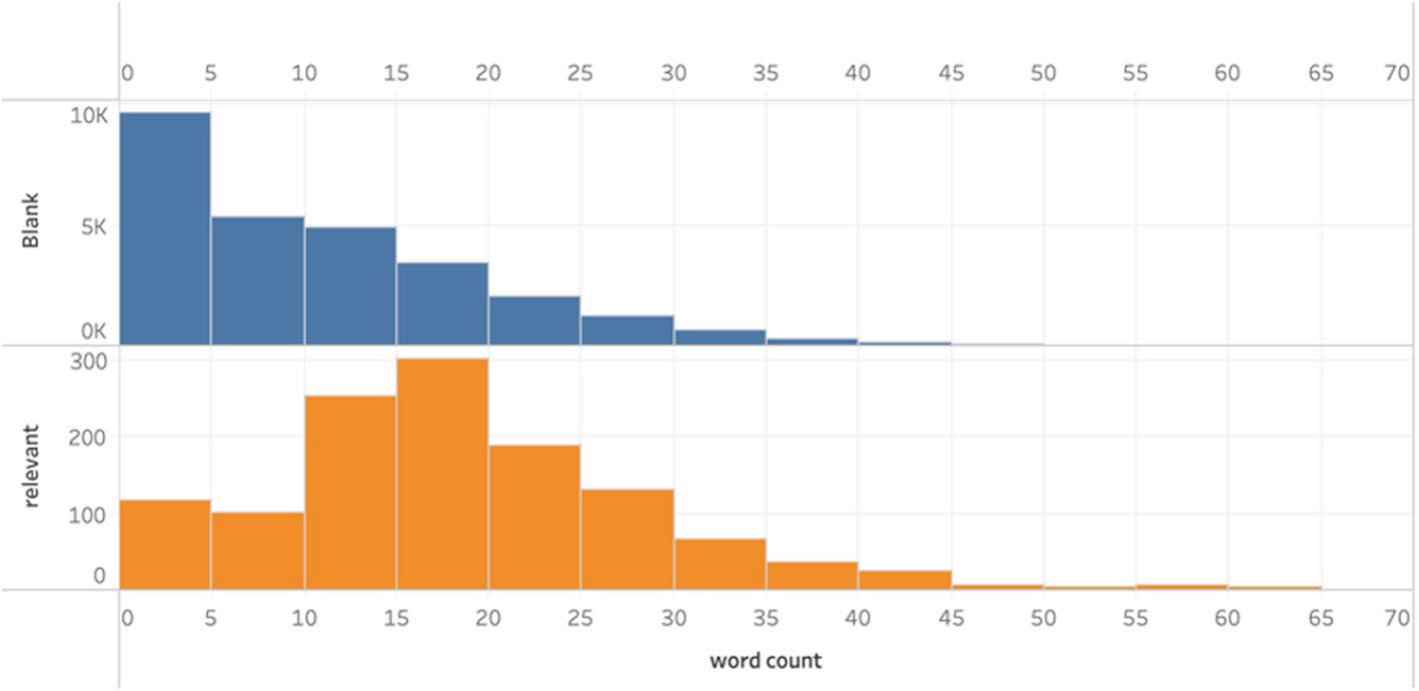
Sentence length distribution

The dataset contained 29,204 sentences. As shown in Fig. 2, this dataset was highly unbalanced. The ratio of blank vs relevant records was 22.4. An analysis on the distribution of the number of words in blank and relevant sentences showed a notable difference between the two classes. Blank class had far more sentences shorter than 5 words (shown in Fig. 3), which created a basis for balancing the dataset. We created a cropped dataset by deleting all sentences with fewer than 5 words, which contained 19,380 sentences, with 1133 relevant records and 18,247 blank records. This decreased the ratio of blank vs relevant to 16.1, but the cropped dataset was still unbalanced. To overcome this issue, we used the Synthetic Minority Over-sampling Technique (SMOTE) to over-sample the relevant class in both the original and cropped dataset.

### Variance in Kappa scores

During the gold standard development stage, Cohen’s Kappa scores were calculated to evaluate the difference between the two annotators. The Kappa scores are summarized in Table 1. Blank entries indicate no sentences were labeled for the question in that round. When there were too few labeled sentences, the kappa score was sometimes zero. Among the permission questions, Q8 and Q9, which concern the commercial use of donor data and recontact, have the highest kappa scores.

**Table 1:** Kappa scores

Permissions	Round 1	Round 2	Round 3	Round 4	Round 5	Mean (SD)
Blank	0.58	0.68	0.62	0.66	0.76	0.66 (0.07)
Q1	0.41	0.29		0.78	0.89	0.59 (0.29)
Q2	0.50	0.59		0.52	0.75	0.59 (0.11)
Q3	0.42	0.71	0.40	0.40	0.59	0.50 (0.14)
Q4	0.07	0.50	0.00	0.00	0.26	0.17 (0.22)
Q5	0.59	0.65	0.79	0.47	0.72	0.64 (0.12)
Q6	0.38	0.66	0.54	0.72	0.73	0.61 (0.15)
Q7	0.59	0.56	0.00		0.88	0.50 (0.37)
Q8	0.75	0.73	1.00	0.67	0.89	0.81 (0.14)
Q9	0.59	0.77	0.80	0.75	0.71	0.72 (0.08)

### Model performance

#### Relevance classification

We first tested RF, linear SVM, polynomial SVM and Gaussian (RBF) SVM models on the classification of relevance. All four models were tested both on the original dataset and the cropped dataset. Their performances on predicting relevance based on the test set are summarized in Table 2, all computations were run on a MacBook Pro (Intel i7) with macOS Catalina. The Linear SVM had the shortest training time and the highest recall, Table 3 summarizes its performances classifying relevant and non-relevant sentences. The cropped dataset generally produced better results than the complete dataset.

**Table 2:** Average model performances for relevance prediction based on test set

	Precision	Recall	F1-score	Training time(s)
RF (complete)	0.76	0.80	0.78	2478
RF (cropped)	0.85	0.76	0.80	1578
Linear SVM (complete)	0.59	0.81	0.61	0.8145
Linear SVM (cropped)	0.69	0.83	0.74	0.4923
Poly SVM (complete)	0.60	0.74	0.63	9314
Poly SVM (cropped)	0.83	0.79	0.81	4312
RBF SVM (complete)	0.61	0.73	0.64	4612
RBF SVM (cropped)	0.59	0.64	0.62	3768

**Table 3:** Linear SVM model performance based on test set

	Precision	Recall	F1-score	Number of sentences
Relevant	0.41	0.72	0.52	215
Blank	0.98	0.94	0.96	3661

### Permission type classification

**Fig. 4:**
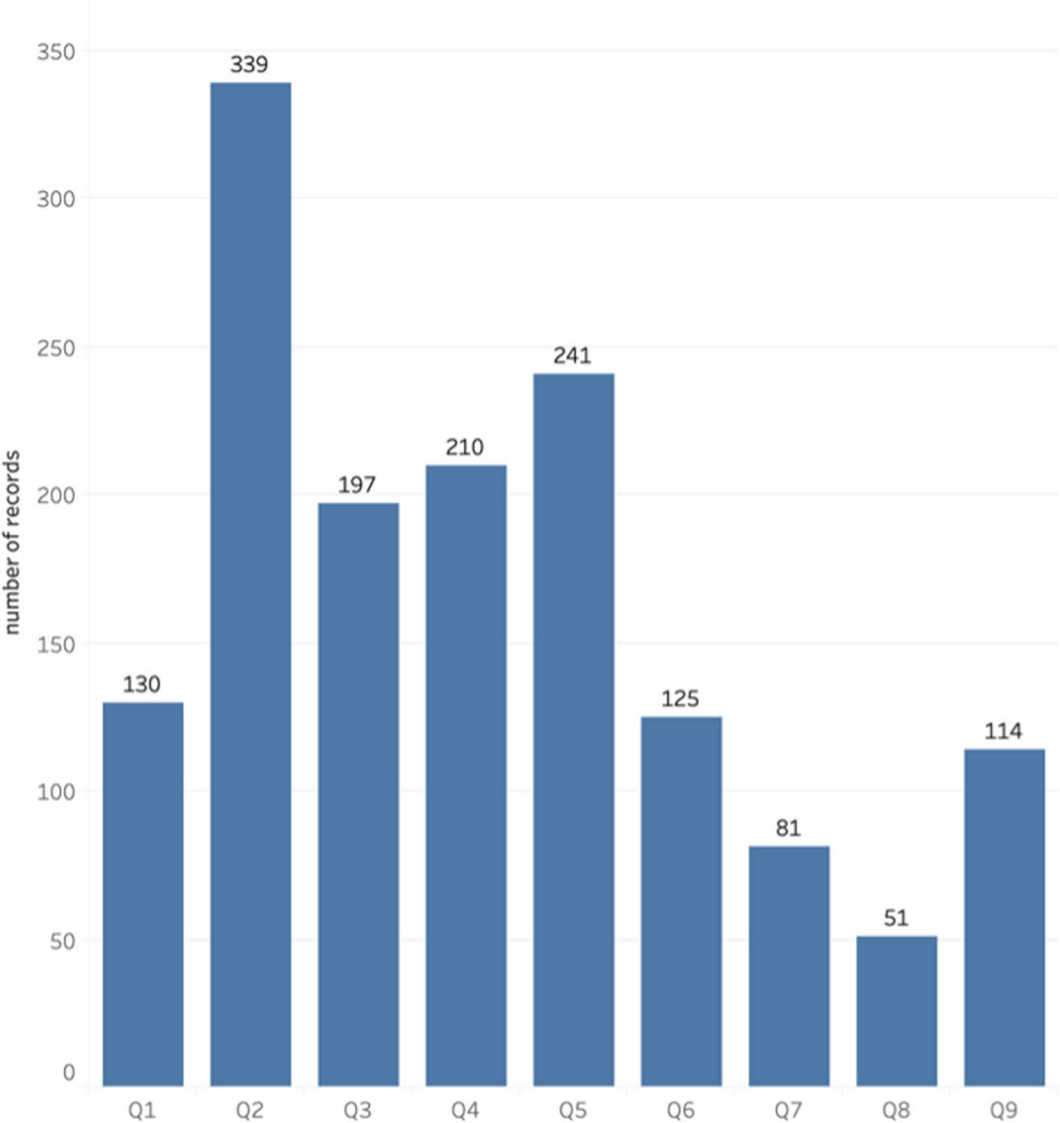
Number of sentences related to each permission question

**Fig. 5:**
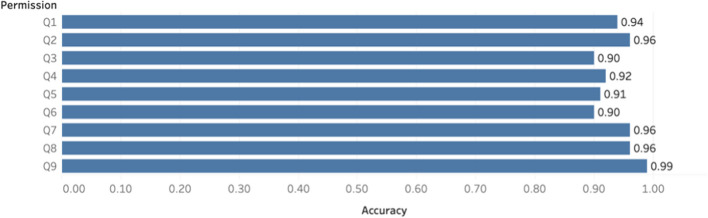
Model accuracies based on permission type

The goal was to use predicted relevant sentences as input, then to classify those into one of the nine permission questions. However, due to the scarcity of records in each permission type (see Fig. 4) and the relatively poor performance of the relevance classification, this was not feasible because the training set would contain too few records for some permission questions. For this reason, the manually annotated gold standard (all sentences manually labeled as relevant in the cropped dataset) was used for training and evaluating the permission type classification. Figure 4 shows the distribution of numbers of sentences related to each question. Based on the result of relevance classification, linear SVM had the highest recall, comparable F-1 score with the best model and was much faster to train. Due to the relatively large numbers of questions to classify, linear SVM was chosen for permission type classification. For each permission question, a binary classification model was built to predict whether a sentence answers a particular permission question. Their performances on the validation set are summarized in Table 4 and Fig. 5. The results show that given a sentence if relevant, our model has a high accuracy in predicting if it answers a specified permission question. For comparison, the F-1 score of one-step classification, which refers to classifying a sentence into a permission question from all sentences in an informed consent document, and two-step classification, classifying only the relevant sentences into a permission question, is shown in Table 5.

**Table 4:** Permission type classification performances

Permission	Label	Precision	Recall	F1-score	Number of sentences
Q1	No	0.97	0.96	0.97	204
	Yes	0.69	0.74	0.71	23
Q2	No	0.94	0.95	0.94	170
	Yes	0.84	0.81	0.82	57
Q3	No	0.93	0.95	0.94	185
	Yes	0.75	0.66	0.70	41
Q4	No	0.94	0.96	0.95	180
	Yes	0.72	0.81	0.76	47
Q5	No	0.92	0.98	0.95	171
	Yes	0.91	0.75	0.82	56
Q6	No	0.95	0.94	0.95	203
	Yes	0.54	0.58	0.56	24
Q7	No	0.99	0.97	0.98	208
	Yes	0.73	0.84	0.78	19
Q8	No	0.97	0.99	0.98	215
	Yes	0.75	0.50	0.60	12
Q9	No	0.99	1.00	1.00	204
	Yes	1.00	0.91	0.95	23

**Table 5:** Average F1-scores comparison

	Q1	Q2	Q3	Q4	Q5	Q6	Q7	Q8	Q9
One step	0.67	0.74	0.65	0.65	0.69	0.64	0.78	0.70	0.68
Two steps	0.84	0.86	0.82	0.86	0.89	0.75	0.88	0.79	0.97

## Discussion

The overall low Kappa scores demonstrate striking difficulty discerning the expression of permissions in informed consent forms. It also illustrates the lack of details for clearly conveying donor permission in current informed consent documents. The ambiguity in the informed consent language translates into the difficulty to build a high-performance NLP model to annotate automatically donor permissions. As shown in Table 1, Q8 and Q9 have the highest kappa scores, showing they are relatively less ambiguous to annotate. This clarity enables the machine learning model to achieve higher accuracy compared to other questions. Among the permission questions, our model performed worst on Q6, “donor data permitted to be used for future research”, compared to the high performance on Q5, “bio-specimen permitted to be used for future research”. This could be due to the challenge in identifying “donor data”, which covers a wide range of information such as patient medical records, genetic data, study results, or any unspecified data. The kappa score for Q6 started low (0.378) but improved over the course of 5 rounds of manual annotation, this is difficult for a “bag-of-words” NLP approach because the words used in these sentences can be very eclectic. As human biospecimens are increasingly being shared across clinical research studies, more standardized informed consent terminology and template should and is being developed. We have developed an initial “Informed Consent Ontology” (ICO) [14, 15] and will expand the ontology on the richness and complexity of real-life informed consent processes.

On the other hand, NLP provides opportunity to show patterns that human fail to recognize. Our current corpus has limited numbers of relevant sentences, which limits the machine learning ability. Our study may serve as a reference for future studies to develop better guidelines for such annotations so higher inter-rate reliability can be achieved. Given a larger annotated corpus or semi-supervised approach, NLP hold great potential to track automatically donor permission.

The complexity of regulatory, privacy and security requirements involved in clinical research present consequential challenges to our ability to build information systems that support sharing of research data and specimens at scale. This study tackles a slice of this challenge by providing insight into the potential difficulties in automating the information sharing workflow in the clinical research setting. Future researchers can implement our findings in their construct of informed consent documents with feasibility of automation in mind. Our study also serves as a foundation and reference point for developing larger scale information systems. This study is, however, limited in its implementation due to the relatively small data set and lack of testing on information retrieval regarding permissions in the regulatory domain.

## Conclusions

To the best of our knowledge, this study is the first attempt to annotate automatically informed consent documents using NLP methods. Even though much room exist for improvement of the machine-learning models, by using a simple and fast algorithm such as linear support vector machine and achieving acceptable results, this study illustrates the possibility for building tools to promote biorepository data regulation, sharing, and decision support. Future research can focus on developing more advanced machine learning or deep learning models to improve the accuracy of classifying permissions contained in informed consent documents.

## Methods

### Corpus development

We collected publicly available informed consent form templates, annotated templates, example documents, instructions and other relevant documents related to informed consent processes used for human subjects research from online resources including websites of Institutional Review Board (IRB), Clinical and translational research groups, Office of Sponsored Program, Office of Research within universities, hospitals, research institutions, biobanks etc. and regulatory agencies. A total of 178 documents including 93 annotated templates, 60 example documents, 10 documents containing instructions and 15 templates were collected. We randomly selected 120 documents for manual review and semantic annotation.

### Permission questions development

A panel of domain experts with Ph.D. or M.S. backgrounds in nursing, clinical research and biomedical informatics identified 9 important permission-related questions this study seeks answers from informed consent forms, broadly covering 4 categories: permission related to future research, permission related to genetic research, permission related to sharing, and permission related to recontact. We finalized the 9 questions through an iterative process of discussion, voting, and test annotation. Table 6 summarizes the finalized questions. Granularity was set at sentence level; boundary was set from first non-white-space character to last punctuation character. The general guidelines are:

- If it’s important to retrieve as evidence with regard to answering the question, annotate the sentence (including sentences that provide important context for content that follows), and considered ‘relevant’. Otherwise, it will be considered ‘blank’.
- Annotate each sentence individually, even if they are part of a sequential group.
- Apply all relevant questions to each sentence.
- If a sentence provides evidence for two or more questions, mark the sentence with tags for each.
- Do not markup text that is clearly information about the study, not specific to what will happen to “you” or the donor.

**Table 6:** Permission questions

Label	Answers to permission questions
Q1	Biospecimens permitted to be shared
Q2	Donor data permitted to be shared
Q3	Biospecimens permitted to be used for genetic research
Q4	Donor data permitted to be used for genetic research
Q5	Biospecimens permitted to be used for future research
Q6	Donor data permitted to be used for future research
Q7	Biospecimens permitted to be used for commercial purposes
Q8	Donor data permitted to be used for commercial purposes
Q9	Donor recontacts permitted

### Gold standard development

Two independent experts with backgrounds in clinical research and biomedical informatics annotated the corpus using the Clinical Language Annotation, Modeling, and Processing (CLAMP) software [10]. We developed the gold standard in a manner of iterative rounds. In the first five rounds, the two domain experts annotated the same sample of informed consent documents. The annotated text was parsed using an in-house developed tool and compared by calculating Cohen’s Kappa scores [11]. After each round, the differences were reconciled, and updates were added to the guideline. 40 documents were annotated in this manner (10 documents did not contain any permission questions), the remaining 80 documents were split equally to be annotated independently (11 documents did not contain permission questions). An example of the annotated text is shown in Fig. 1.

### NLP model development

We approached the problem of predicting whether a sentence in an informed consent document contains answers to the permission questions described as a supervised classification task. The annotated corpus was parsed and transformed into one-hot encoding format, with each sentence in a row and each permission question as a categorical variable. Sentences without permission labels were categorized as “blank”. We tokenized each sentence using spaCy package[12] in Python, removed punctuations and stop words, then further removed single characters, special characters, and multiple spaces or newlines. We converted the processed sentences into term frequency and inverse document frequency (TF-IDF) features using Python’s Scikit-Learn library[13]. Because of the dominance of “blank” sentences in the corpus, we broke down the classification task into two sub-tasks: (1) classification of relevance, defined as being “blank” or not, and (2) classification of permission type, defined as any one of the permission questions. For classifying relevance, the complete dataset was split into training and testing set by a ratio of 8:2. Then we used packages from Scikit-Learn to classify each sentence into either “blank” or relevant. For classifying permission type, all relevance sentences were extracted then split into training and testing set by a ratio of 8:2. We tested four machine learning algorithms: random forest (RF), polynomial support vector machine (SVM), linear SVM and Gaussian SVM.

## Data Availability

Due to privacy concerns, the annotated data set is not publicly available at this time but may be released in the future with data usage agreements upon request.

## Abbreviations

NLP: Natural Language Processing
RF: Random Forest
SVM: Support Vector Machine
SMOTE: Synthetic Minority Over-sampling Technique
TF-IDF: Term Frequency and Inverse Document Frequency
CLAMP: Clinical Language Annotation, Modeling, and Processing
IRB: Institutional Review Board
ICO: Informed Consent Ontology
RBF: Radial Basis Function
